# Transitioning from video-assisted to robotic-assisted anatomical pulmonary resection: outcomes from 340 cases in an Australian centre

**DOI:** 10.3389/fonc.2025.1647273

**Published:** 2025-09-30

**Authors:** Daniel Shell, Mohd Firdaus, Natcha Bunwatcharaphan, Jacob Gordon, Manoras Chengalath, Cheng-Hon Yap

**Affiliations:** ^1^ Department of Cardiothoracic Surgery, Barwon Health, Geelong, VIC, Australia; ^2^ School of Medicine, Deakin University, Geelong, VIC, Australia; ^3^ Department of Epidemiology and Preventive Medicine, Monash University, Melbourne, VIC, Australia

**Keywords:** thoracic surgery, thoracic oncology, rats, VATS, lobectomy, segmentectomy, lung cancer

## Abstract

**Background:**

Robotic-assisted thoracoscopic surgery (RATS) is emerging as a technically advanced alternative to video-assisted thoracoscopic surgery (VATS) for anatomical pulmonary resection. While its potential benefits include enhanced visualisation and precision, real-world outcome data remain limited—particularly within the Australian healthcare setting. This study evaluates short-term clinical and oncologic outcomes during the transition from VATS to RATS in a single-centre thoracic surgery practice.

**Methods:**

We conducted a retrospective cohort study of 340 consecutive thoracoscopic anatomical lung resections (segmentectomy, lobectomy, or pneumonectomy) performed by a single surgeon between July 2012 and February 2025 in Geelong, Australia. Short-term outcomes from the first 170 patients treated with RATS during the surgeon’s initial robotic experience were compared with those of a historical cohort of 170 VATS patients.

**Results:**

Baseline demographics were similar, although the RATS group included a higher proportion of obese patients and segmentectomies. Both groups demonstrated low complication and mortality rates. RATS was associated with significantly higher lymph node yield (mean 11 vs 8 nodes, p<0.001) and shorter postoperative pleural drainage duration (2.2 vs 3.8 days, p<0.001). Hospital length of stay was reduced by more than two days in the RATS group (4.4 vs 6.4 days, p<0.001). Operative time and rates of conversion, upstaging, and readmission were comparable between groups.

**Conclusion:**

RATS was safely introduced without increased operative time or complication rates and was associated with improved lymphadenectomy and enhanced postoperative recovery. As the largest Australian comparison of RATS and VATS to date, these findings support the integration of robotic pulmonary resection into standard thoracic surgical practice.

## 1 Introduction

Anatomical pulmonary resection—comprising pneumonectomy, lobectomy, and segmentectomy—remains the cornerstone of curative treatment for surgically resectable non-small cell lung cancer (NSCLC). The introduction of video-assisted thoracoscopic surgery (VATS) marked a significant advancement in thoracic oncology, offering reductions in perioperative pain, morbidity, and mortality, while providing equivalent long-term oncological outcomes to standard thoracotomy (1). Building upon these principles of minimally invasive surgery, robotic-assisted thoracoscopic surgery (RATS) has emerged as a potential further refinement, offering enhanced three-dimensional visualization, greater instrument articulation, and improved operative precision (2). In addition, recent reviews have highlighted that RATS offers ergonomic advantages through its console-based design, which can reduce musculoskeletal strain and fatigue for the operating surgeon, supporting sustained performance during complex cases (3).

Although RATS was first described in 2002, its adoption in Australia has been comparatively gradual and limited, with most procedures concentrated within high-volume metropolitan centres (4). International evidence has confirmed that RATS is safe and feasible, yet its superiority over VATS remains the subject of ongoing debate. Some meta-analyses report advantages in perioperative outcomes such as reduced blood loss, shorter length of stay, and higher lymph node yields, while others find largely equivalent results between RATS and VATS (5, 6). A recent comprehensive review by Zhang and colleagues further underscored this controversy, concluding that although RATS is a credible minimally invasive alternative, definitive evidence of consistent superiority over VATS remains limited (7). Consequently, comparative data in the Australian setting remain scarce, and real-world studies such as ours are important in clarifying how RATS performs in practice.

Aiming to broaden the accessibility of RATS within the Australian healthcare landscape, a robotic thoracic surgery program was established in 2019 in Geelong, Victoria, Australia by a surgeon already experienced in VATS lobectomy. Since implementation, the surgeon has transitioned to performing all anatomical resections robotically. This study captures the outcomes observed during the surgeon’s initial experience with RATS during the learning curve phase, and compares these to a historical cohort of patients treated with VATS. In doing so, we aim to evaluate the short-term clinical and oncologic outcomes during the transition from VATS to RATS within a real-world, regional Australian context.

## 2 Method

### 2.1 Study design

We conducted a retrospective observational cohort study of all thoracoscopic anatomical pulmonary resections performed by a single surgeon at a regional centre in Geelong, Victoria, Australia. This cohort includes every consecutive thoracoscopic anatomical lung resection—via either video-assisted (VATS) or robotic-assisted (RATS) approach—performed by this surgeon from the commencement of their thoracoscopic practice in July 2012 through to February 2025. Patients were operated on at two centres: University Hospital Geelong, and St John of God Geelong Hospital. A total of 340 cases were identified. Patients undergoing wedge resections or non-pulmonary thoracic procedures were excluded. While the majority of cases were performed for suspected non-small cell lung cancer, all patients were included in the completed data analysis regardless of final histopathological diagnosis.

### 2.2 Data analysis and statistical methods

Clinical data was collected retrospectively from patient electronic medical records and online results portal. Lymph node data was extracted from the formal histopathology report and final staging was evaluated according to the AJCC (American Joint Committee on Cancer) Cancer Staging Manual (TNM) 7th edition (8). All data were structured using the ANZTHOR (Australian and Aotearoa New Zealand Thoracic clinical quality registry) thoracic surgery database format (9) to facilitate future benchmarking and national comparison.

The study was approved by the Barwon Health Research Ethics Committee (HREC/97887/VICBH-2023-376758(v1)), with a waiver of individual patient consent due to the low-risk, retrospective design. Data cleaning and statistical analyses were performed using R software (version 4.3.0, R Core Team, Vienna, Austria). Continuous variables were compared using the Mann–Whitney U test, while categorical variables were analysed using the Pearson’s chi-squared test. A two-sided p-value 0.05 was considered statistically significant.

### 2.3 Operative technique

A VATS approach was predominantly utilised in the surgeon’s early years of practice. VATS anatomical resection was performed using a standard multi-portal anterior approach, typically employing three ports. Surgical technique was tailored to tumour location, size, and patient anatomy, in accordance with established institutional protocols.

From mid-July 2019 all subsequent anatomical resections were undertaken via a RATS approach. RATS was conducted using the da Vinci Xi robotic system (Intuitive Surgical, USA) with P8 software. The operative setup most closely resembled the RPL-4 technique. Four robotic ports were inserted spaced approximately 8–10 cm apart. In most cases three 8 mm ports were placed in the 8th intercostal space and one anterior 12 mm port was sited in the 7th intercostal space near the costal margin. For left upper lobectomies and complex segmentectomies, a second 12 mm port (instead of a 8mm port) was introduced to allow a second stapler option. An additional 12 mm assistant port was placed in the 9th intercostal space to allow for endoscopic suction, retraction, and specimen extraction. All ports were sealed, and the hemithorax insufflated with carbon dioxide (5–8 mmHg) to improve operative visibility and facilitate smoke clearance.

For all suspected malignant cases, we attempted to perform a systematic mediastinal lymph node dissection in accordance with international oncological standards. For right-sided resections this includes dissection of stations 2R, 4R, 7, 8, 9 and 10, and for left-sided resections stations 5, 6, 7, 8, 9 and 10. However, in practice, complete clearance was occasionally limited by anatomical constraints, particularly in VATS procedures.

In both approaches, an intercostal catheter drain (ICC) was routinely placed through one of the existing port sites and connected to suction at –20 cm H_2_O for at least the first few postoperative hours. Drains were removed when there was no detectable air leak on forced expiration/cough and fluid drainage was <200 mL over 24 hours. Most patients received a perioperative regional anaesthetic block—either erector spinae or paravertebral catheter—to facilitate post-operative recovery. Although elements of enhanced recovery were gradually introduced over the study period, no formal ERAS (Enhanced Recovery After Surgery) program was in place in our institution.

## 3 Results

### 3.1 Demographics

The final cohort consisted of 340 patients, with 170 undergoing VATS and 170 undergoing RATS. Patient demographics and comorbidities are summarised in **Table 1**. The two groups were comparable in terms of age, sex distribution, pulmonary comorbidities, and cardiovascular disease. However, significant differences were observed in body mass index (BMI), smoking status, performance status (ECOG), and history of prior cardiothoracic surgery. Mean BMI was higher in the RATS group (28.9 vs 27.7 kg/m², p=0.007), and the proportion of patients classified as obese (BMI ≥30) was significantly greater in the RATS cohort (42% vs 24%, p=0.001).

**Table 1:** Patient demographics and operative data.

All patients	RATS (n=170)	VATS (n=170)	p-value
Age (years)	68.0 (9.9)	66.6 (10.5)	0.14
Male Sex	42%	48%	0.2
BMI	28.9 (5.10	27.7 (5.7)	**0.007**
BMI ≥ 30	42%	24%	**0.001**
Smoking History			**0.013**
Never Smoker	39%	25%	
Ex/Current smoker	61%	75%	
Pulmonary Comorbidities	30%	36%	0.2
Cardiovascular Comorbidities	29%	21%	0.078
ECOG Status			**0.033**
0	79%	69%	
1	19%	31	
2	1%	1%	
Prior Cardiothoracic Surgery	11%	4%	**0.022**
Type of Resection			
Segmentectomy	35%	8%	**<0.001**
Lobectomy	64%	90%	**<0.001**
Bi-lobectomy	1%	2%	0.6
Pneumonectomy	1%	1%	>0.9
Final Histopathology			
NSCLC	79%	84%	0.3
Pulmonary Metastasis	10%	9%	0.7

BMI, Body Mass Index; ECOG, Eastern Cooperative Oncology Group; NSCLC, Non-Small Cell Lung Cancer. Bold signifies statistical significance (i.e. p<0.005).

### 3.2 Operative details

Operative details are outlined in **Table 1**. Lobectomy was the most common procedure in both groups, accounting for 90% of VATS and 64% of RATS cases (p<0.001). However, the RATS cohort included a significantly higher proportion of segmentectomies (35% vs 8%, p<0.001). Distribution of resections by lobe was similar across groups, and one pneumonectomy was performed in each cohort.

### 3.3 Oncological outcomes

A total of 276 patients were diagnosed with non-small cell lung cancer (NSCLC) on final histopathology, comprising 142 patients in the VATS group and 134 in the RATS group (**Table 2**). The remaining cases were treated for either metastatic disease or benign conditions. Adenocarcinoma was the predominant histological subtype across both groups, followed by squamous cell carcinoma and carcinoid tumours. The histological distribution was similar between cohorts and broadly reflects national epidemiological data for lung cancer in Australia (10).

**Table 2:** Oncological data.

Confirmed NSCLC	RATS (n = 134)	VATS (n = 142)	p-value
Clinical N Stage			0.3
0	90%	94%	
1	6%	4%	
2	4%	1%	
Final TNM Stage			
Stage 1 (IA – IB)	67%	68%	0.8
Stage 2 (IIA – IIB)	15%	21%	0.2
Stage 3 (IIIA – IIIC)	18%	9%	**0.033**
Stage 4 (IVA – IVB)	0%	1%	0.5
Histology			
Adenocarcinoma	69%	64%	0.4
Squamous Cell Carcinoma	19%	23%	0.4
Carcinoid or Other	12%	12%	>0.9
Maximum Tumour Diameter	32.4 (23.6)	31.2 (22.7)	0.7
Incomplete (R1) Resection	1%	1%	>0.9
Total Lymph Node Stations Sampled	6 (1)	4 (1)	**<0.001**
Hilar Stations Sampled	2 (1)	1 (1)	**<0.001**
Mediastinal Stations Sampled	4 (1)	3 (1)	**<0.001**
Total Lymph Node Yield	11 (6)	8 (5)	**<0.001**
Hilar Lymph Nodes	4 (2)	3 (2)	**<0.001**
Mediastinal Lymph Nodes	7 (5)	5 (4)	**<0.001**
Surgical Up-stage	10%	18%	0.088

NSCLC, Non-Small Cell Lung Cancer; TNM, Tumour, Node and Metastasis. Bold signifies statistical significance (i.e. p<0.005).

Pre-operative clinical nodal staging did not differ significantly; however, there was a trend toward greater N2 involvement in the RATS cohort (4% vs 1%), which corresponded with a significantly higher proportion of pathological stage III disease in this group (18% vs 9%, p=0.033).

All patients with suspected malignancy underwent formal mediastinal lymph node dissection. The RATS group had a significantly higher mean number of mediastinal (N2) stations sampled (4 vs 3, p<0.001) and a greater total lymph node yield (11 vs 8, p<0.001). Despite the increased nodal assessment, there was no significant difference in the rate of pathological upstaging between groups (RATS 10% vs VATS 18%, p=0.088). Complete microscopic (R0) resection was achieved in 99.4% of patients overall, and no macroscopically incomplete (R2) resections were observed.

### 3.4 Short-term outcomes

Postoperative outcomes are summarised in **Table 3**. Two early mortalities occurred in the cohort (0.59%), with one in each surgical group. The VATS-related death occurred in an immunosuppressed patient who had received neoadjuvant chemotherapy for N2 disease and post-operatively developed a broncho-pleural fistula, ultimately succumbing to sepsis. The RATS-related death was attributed to an acute exacerbation of pre-existing interstitial lung disease, resulting in refractory respiratory failure. All other patients were followed for at least 90 days, with no additional deaths recorded.

**Table 3:** Complication data and clinical outcomes.

All patients	RATS (n=170)	VATS (n=170)	p-value
Total Operative Time	166.2 (56.5)	172.7 (48.7)	0.065
Any Complication (CTCAE Score ≥3)	12%	15%	0.4
PRBC Transfusion	2%	2%	>0.9
Return to Theatre	2%	5%	0.2
Unplanned ICU Admission	2%	2%	>0.9
30-day Mortality	1%	1%	>0.9
Conversion to Thoracotomy	2%	5%	0.13
Discharged with Post-operative ICC	0%	2%	0.3
Post-operative ICC (days)	2.2 (3.1)	3.8 (4.4)	**<0.001**
Length of Stay (days)	4.4 (3.6)	6.4 (5.2)	**<0.001**
Readmission within 30 days	7%	14%	**0.034**

CTCAE, Common Terminology Criteria for Adverse Events; PRBC, Packed Red Blood Cell; ICU, Intensive Care Unit; ICC, Intercostal Catheter. Bold signifies statistical significance (i.e. p<0.005).

Postoperative complications were graded according to the Common Terminology Criteria for Adverse Events (CTCAE), with a score of ≥3 indicating the need for an invasive intervention (e.g., chest drain, bronchoscopy, or reoperation). There were no statistically significant differences between groups in the rate of major complications (CTCAE ≥3), blood transfusion, reintubation, or unplanned intensive care admission. Return to theatre and conversion to thoracotomy occurred less frequently in the RATS group, although these differences did not reach statistical significance.

Significant differences emerged in time-related postoperative recovery metrics. ICCs were removed earlier in the RATS group (mean 2.2 vs 3.8 days, p<0.001), and the mean length of hospital stay was reduced by over two days (4.4 vs 6.4 days, p<0.001). No RATS patients were discharged with an ICC *in situ*, compared to 2% of patients in the VATS group who were discharged with a drain due to prolonged air leak. Additionally, 30-day readmission rates for any cause were lower in the RATS cohort (9% vs 14%, p=0.034).

### 3.5 Operative time

As an additional marker of operative efficiency and workflow, total operative time was recorded for all cases. To ensure consistency between groups, operative time was defined from surgical time-out to final application of dressings. For RATS procedures, this measurement included draping and docking of the robotic system, thereby reflecting the performance of both the surgeon and the theatre team.

**Figure 1:**
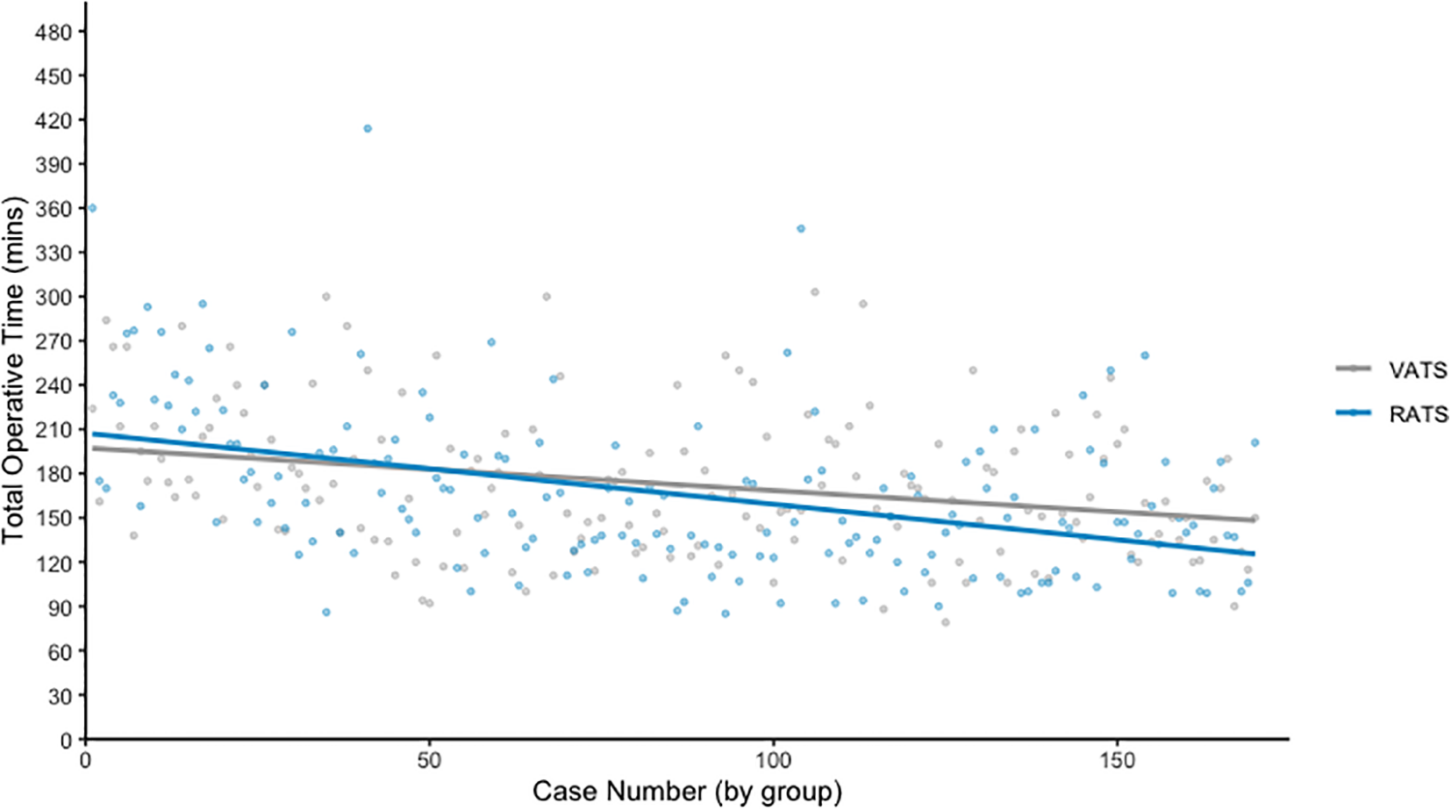
Graph of total operative time.

**Figure 1** plots operative time against case number for each surgical modality, with a line of best fit demonstrating trends over time. As expected, RATS procedures initially required longer operative times during the early adoption phase. However, this duration decreased progressively with experience. Over the entire series, there was no statistically significant difference in mean operative time between the groups (RATS: 166.2 minutes vs VATS: 172.7 minutes, p = 0.065).

## 4 Discussion

This single-surgeon cohort provides a practical reference point for thoracic surgeons considering the adoption of robotic-assisted techniques into their existing minimally invasive practice. The study is distinct in that it includes the first 100 anatomical pulmonary resections performed by the surgeon using both VATS and RATS, thereby capturing the learning curve associated with each modality. As such, the outcomes presented reflect real-world, early-experience results that may be particularly informative for centres seeking to initiate a robotic thoracic program.

As a non-randomised, observational study, differences in baseline characteristics between groups are expected and must be interpreted with caution. One of the most notable disparities was the higher proportion of clinically obese patients in the RATS cohort. This likely reflects a referral bias, whereby patients with higher BMI—who may present greater technical challenges—were preferentially directed toward the robotic approach due to its enhanced exposure, stability, and precision. At our center, all surgical cases are first reviewed in a multidisciplinary meeting, and referrals for RATS in obese patients were frequently driven by perceived procedural advantages. This trend aligns with findings from Seder et al., whose large-scale database analysis of over 8,000 obese patients demonstrated reduced conversion to thoracotomy and shorter length of stay among those undergoing RATS compared to VATS (11).

Although lobectomy remained the predominant procedure across both groups, a notable transition toward sublobar resection was observed in the RATS cohort, with a significantly higher rate of segmentectomies. Importantly, this shift did not lead to increased complication rates or prolonged air leak, despite the greater technical demands of segmental dissection. The increase in segmentectomies coincided with the latter half of the study period, reflecting both growing surgical confidence in the robotic platform and emerging evidence supporting segmentectomy in selected patients with early-stage NSCLC (12). While this study was not powered to detect differences in segmentectomy-specific outcomes, our findings are consistent with those of Zhang et al., who reported no significant differences between RATS and VATS for segmentectomy in a large multi-institutional cohort (13).

We found that the RATS cohort had a significantly greater number of lymph node stations sampled (both mediastinal and intra-pulmonary) and total lymph nodes retrieved. This likely reflects the technical advantages of the robotic platform, including enhanced visualisation, articulation, and access to confined mediastinal spaces. At our centre, lymphadenectomy is routinely performed at the beginning of RATS procedures, as this tends to improve access and mobilization for subsequent anatomical resection. Nonetheless, the increase in nodal yield did not result in a significantly higher rate of surgical nodal upstaging. This finding is consistent with larger retrospective series, such as those by Hennon et al. and Merritt et al., which also reported greater lymph node harvest with RATS but no clear difference in staging outcomes (14, 15). These results largely reflect the sensitivity and accuracy of current methods of clinical nodal staging in NSCLC, such as the use of Positron Emission Tomography and endobronchial ultrasound guided nodal sampling.

Importantly, the introduction of RATS did not result in an increase in perioperative complications, despite capturing the early learning curve of the surgical team. Rates of return to theatre, transfusion, unplanned ICU admission, and 30-day mortality were low and comparable between groups. Operative times were initially longer in RATS cases, as expected during the early adoption phase due to unfamiliar workflows and surgeon experience. However, these durations improved steadily over time, and when analysed across the entire cohort, the mean operative time was not significantly different from VATS. These results support previous findings that robotic anatomical resection can be introduced safely and effectively, even in relatively low-volume centres, without compromising patient outcomes during the learning period.

The most clinically meaningful difference between groups was observed in post-operative recovery, particularly intercostal catheter duration and hospital length of stay. ICCs were removed significantly earlier in RATS patients, and average hospitalisation was reduced by more than two days. Notably, no RATS patients required discharge with a drain *in situ*, in contrast to a small but meaningful proportion in the VATS cohort. This improvement likely reflects reduced tissue trauma and more precise dissection with the robotic system. Although our study did not explicitly capture post-operative pain scores, this is certainly a secondary motivation for transitioning to RATS resections. Prospective studies such as those by Catelli et al. (16) and Huang et al. (17) have surmised that the reduced surgical inflammatory insult of RATS clinically manifests in reduced post-operative pain and this might in turn contribute to a reduced length of stay. In the context of rising healthcare costs and increasing demand for inpatient beds these findings have practical implications. Shorter length of stay not only improves patient experience but may also help offset the capital and consumable costs of robotic surgery. These data are supported by early findings from the RAVAL trial, which demonstrated that despite higher direct procedural costs for RATS, the approach achieved cost-neutrality at 12 weeks when factoring in reduced length of stay, lower complication rates, and faster return to usual activities (18).

We acknowledge several limitations inherent with the study design. This study is limited by its retrospective, observational design, which precludes randomisation and introduces the potential for selection bias. The use of a historical VATS cohort introduces the possibility of temporal confounding, including changes in referral patterns, surgical decision-making, and perioperative care over time. Additionally, this series represents the experience of a single surgeon, which enhances internal consistency but may limit external generalisability. The sample size, while among the largest Australian cohorts to date, may still be underpowered to detect subtle differences in uncommon outcomes or to perform robust subgroup analyses. Finally, this study was only designed and powered for short-term results, with longer-term outcomes such as overall survival (OS) or disease-free survival (DFS) intended to be the target of future studies.

## 5 Conclusion

To our knowledge, this study represents the largest Australian comparison of RATS and VATS for anatomical pulmonary resection and offers a unique perspective on the transition between these two approaches within the same surgical practice. The transition to RATS in our centre was achieved without an increase in operative time or complication rates, and was associated with greater lymph node yield, earlier chest drain removal, and a shorter hospital stay. These differences were observed despite a higher proportion of technically complex cases, including segmentectomy and operations in higher-risk patients, in the RATS group. These findings suggest that the transition to RATS is not only feasible but may actively enhance certain aspects of surgical care. Our experience highlights how robotic adoption can drive both technical refinement and workflow evolution, even in the early stages of program development.

## Data Availability

The raw data supporting the conclusions of this article will be made available by the authors, without undue reservation.
